# First and Second Reports of the Commissioners for Inquiring into the State of Large Towns and Populous Districts

**Published:** 1845-09

**Authors:** 


*First and Second Reports of the Commissioners for inquiring into the state of Large Towns and Populous Districts.* Presented to both Houses of Parliament by command of her Majesty. Fol. Lond., 1844, 1845.

